# Periodontal disease in cats under primary veterinary care in the UK: frequency and risk factors

**DOI:** 10.1177/1098612X231158154

**Published:** 2023-03-13

**Authors:** Dan G O’Neill, Alyx Blenkarn, Dave C Brodbelt, David B Church, Alix Freeman

**Affiliations:** 1 Pathobiology and Population Sciences, The Royal Veterinary College, Hawkshead Lane, North Mymms, Hatfield, UK; 2 Clinical Science and Services, The Royal Veterinary College, Hawkshead Lane, North Mymms, Hatfield, UK; 3 Department of Dentistry, Oral and Maxillofacial Surgery, Eastcott Referrals, Swindon, UK

**Keywords:** Breed, electronic patient record, primary care, purebred, VetCompass

## Abstract

**Objectives:**

Periodontal disease is a multifactorial inflammatory disease that can have major welfare implications in cats. This study aimed to report the frequency and demographic risk factors of periodontal disease and to explore associations with common comorbid disorders in cats in the UK.

**Methods:**

The study included a random sample of 18,249 cats obtained from 1,255,130 cats under primary care during 2019 from clinics participating in the VetCompass programme. All disorders recorded during 2019 were extracted and reported. Risk factor and comorbid disorder analysis used multivariable logistic regression modelling.

**Results:**

Periodontal disease had a 1-year period prevalence of 15.2% (95% confidence interval [CI] 14.72–15.76). Breeds with the highest prevalence included Siamese (18.7%, 95% CI 12.24–26.72) and Maine Coon (16.7%, 95% CI 11.37–23.18). The median age of cats with periodontal disease (9.47 years, interquartile range [IQR] 5.96–12.97) was higher than for cats without periodontal disease (4.94 years, IQR 1.95–9.51; *P* <0.001). Increasing adult body weight, increasing age and sex–neuter status were significantly associated with rising odds of periodontal disease. Cats with periodontal disease had a higher median count of comorbid disorders per individual cat (3, IQR 2–4, range 1–14) than cats without periodontal disease (1, IQR 0–2, range 0–15; *P* <0.001). Cats with periodontal disease had 1.79 times the odds (95% CI 1.62–1.99, *P* <0.001) of diagnosis with at least one comorbid disorder disease than cats without periodontal disease.

**Conclusions and relevance:**

Periodontal disease is the most common specific diagnosis in cats and is confirmed as a leading health issue in cats. Ageing is identified as the strongest predictor of periodontal disease risk in cats, suggesting the potential for increasing health gains from emphasis on dental care and health in cats as they age. The study offers evidence on a close link between periodontal disease and reduced overall health in cats.

## Introduction

Periodontal disease is a multifactorial inflammatory disease that is reportedly common in domestic cats.^1^ The aetiology involves a complex interaction between subgingival plaque bacteria and the host immune response. In consequence, periodontal disease is characterised by inflammation resulting from microbial by-products such as lipopolysaccharide endotoxins, organic acids, protein toxins and chemotactic peptides. In turn, these stimulate the host immune system to release cytokines such as interleukin (IL)-1 beta, IL-8, prostaglandins and tumour necrosis factor alpha.^2,3^ Gingivitis, the earliest stage of periodontal disease, does not cause tissue destruction and is potentially reversible with professional intervention. Gingivitis need not necessarily progress to periodontitis. Periodontitis is inflammation that results in the gradual destruction of the periodontal tissues comprising the gingiva, periodontal ligament, cementum and alveolar bone, and is not easily reversible.^4,5^

The reported prevalence of periodontitis in cats ranges between 13.9% and 96%,^1,6–8^ with substantial variability depending on the criteria applied for diagnosis and the cohort demographics. Although periodontitis is the most common clinical finding during feline consultations in general practice,^1,9^ it is often overlooked as a source of pain in cats.^10–12^ Recognition of oral pain is challenging because cats are capable of masking overt signs of oral discomfort.^13^ Although many canine studies report risk factors for periodontal disease that include breed, age, insurance status, age, neuter status and body weight,^14–16^ comparable studies are rarer in the feline literature. A study of healthy cats in a colony in France reported breed disposition for periodontitis in Persian, Maine Coon, Burmese, Bengal, Somali and Bobtail cats.^7^ However, several other studies have failed to identify differing prevalence of periodontitis between pure- and mixed-breed cats.^6,17^ Sex and neuter status do not appear to be associated with the risk of periodontal disease in cats,^7^ and the prevalence of periodontal disease in brachycephalic cats^17^ is comparable to results obtained in studies of mesaticephalic cats.^6,7^ No consensus has been achieved regarding ageing as a risk factor for periodontal disease in cats, with results from previous studies varying considerably.^6,7,9,18^

In dogs, a link has been reported between periodontal disease and kidney disease,^19,20^ liver disease,^19,21^ atrioventricular valve pathology,^19^ endocarditis, hypertrophic cardiomyopathy and heart murmur.^22^ There is a lack of studies investigating similar correlations in cats. An association between periodontal disease and the risk of developing chronic kidney disease (CKD) has been reported, with cats with >25% alveolar bone loss showing the greatest risk of developing CKD.^23^ Moderate/severe dental disease has also been shown to be a risk factor for the development of azotaemia in cats.^24^ A greater understanding of the risk factors for, and comorbid relationships between, a broad range of common disorders within a species can facilitate earlier detection in at-risk patients by guiding recommendations for surveillance and monitoring.^25^

Not only can periodontal disease affect the overall health and longevity of cats, but it can also affect the animal’s quality of life and interactions with the owner,^10,26^ making early diagnosis imperative to protect patient welfare.^27^ With a perspective of strengthening the evidence base on periodontal disease in the wider population of UK cats, this study aimed to use anonymised veterinary clinical data from the VetCompass programme^28^ to report the frequency and demographic risk factors of diagnosis with periodontal disease of cats under primary veterinary care in the UK during 2019. Given the value of deeper understanding of aetiological pathways associated with periodontal disease, the study placed special focus on exploring the associations between periodontal disease and common comorbid disorders. These results could assist veterinary practitioners and owners in better understanding and predicting periodontal disease, and in identifying opportunities to improve the health of cats. In line with evidence from studies in dogs,^19,20,22^ we hypothesised that cats with periodontal disease have a higher probability of renal and cardiac conditions as comorbid diagnoses than cats not diagnosed with periodontal disease.

## Materials and methods

The study population included all cats under primary veterinary care at clinics participating in the VetCompass programme during 2019. Cats under veterinary care were defined as those with at least one electronic patient record (EPR; free-text clinical note, treatment or body weight) recorded in 2019. VetCompass collates de-identified EPR data from primary care veterinary practices in the UK for epidemiological research.^28^ Relevant data fields available to VetCompass researchers include a unique animal identifier along with veterinary group identifier, species, breed, date of birth, sex and neuter status, along with clinical information from free-form text clinical notes, summary diagnosis terms^29^ and treatment (with relevant dates).

The study used a retrospective cohort design that followed anonymised clinical records over time. Sample size calculations estimated that 18,058 cats would need to be sampled to report the prevalence of periodontal disease, assuming 13.9% occurrence,^1^ with 0.5% margin of error at a 95% confidence level from a population of 10,000,000 cats in the UK.^30,31^ Ethical approval was obtained from the Royal Veterinary College’s Social Science Ethical Review Board (reference number SR2018-1652).

The study design, data extraction and analysis methods followed previously published methods.^1,16,32^ A random sample of 18,249 cats was obtained from the overall sampling frame of all cats under veterinary care in 2019; all information in the EPR relating to 2019 was manually reviewed to extract the most definitive diagnoses recorded for all disorders that existed in 2019.^16^ EPRs were accessed in the VetCompass online user interface (www.vetcompass.org), and the manual review process was carried out by six final-year veterinary undergraduate students under the direct supervision of the lead author (DGON). Each disorder event was followed over time in the cohort data source to identify the most definitive diagnosis term recorded. Every distinct disorder with evidence for existence within the clinical records was coded to the most precise diagnostic or descriptive term available in the VeNom coding system.^29^ The clinical records available for review included all clinical notes in the practice management system relating to each cat (including but not limited to consultations, house visits, telephone calls, surgical procedures and non-diarised events), as well as all information on any services or items sold at any time for each cat.Disorders described within the clinical notes using presenting sign terms (eg, ‘vomiting’ or ‘vomiting and diarrhoea’) but without a formally recorded clinical diagnostic term were included using the first sign listed (eg, vomiting). Data on elective (eg, neutering) or prophylactic (eg, vaccination) clinical events themselves were not recorded, but any disorders identified during such clinical examinations were included. No distinction was made between pre-existing and incident disorder presentations. For the purposes of analysis, all cats that did not receive a diagnosis with periodontal disease in 2019 were considered as not being a periodontal disease case.The case definition for periodontal disease required evidence in the clinical records that periodontal disease itself or a component disorder (eg, gingivitis, periodontitis, gingival recession and loose teeth) existed as a clinical condition at some point in 2019.^33^ Evidence of only minor calculus or tartar was recorded separately as ‘dental disease’. The clinical decision-making process was at the discretion of the attending veterinary surgeons. Following data checking for internal validity and cleaning in Microsoft Excel 2018, analyses were conducted using Stata Version 16. Breed descriptive information entered by the participating practices was cleaned and mapped to a VetCompass breed list derived and extended from the VeNom Coding breed list.^29^ Breeds represented by >100 cats in the study sample were included individually within a ‘breed’ variable. A ‘purebred’ variable categorised cats of recognisable breeds as ‘purebred’ and cats recorded as mixes of breeds as ‘crossbred’. Sex and neuter status were defined by the final available EPR value. Adult body weight was defined as the mean of all available body weight (kg) values recorded for each cat after reaching 9 months of age. Age (years) was defined at 31 December 2019 as the final date by which each cat in the cohort was classified as either a case or a non-case for each disorder. Body weight and age were categorised for risk factor analysis. ‘Veterinary group’ described the originating larger groups of veterinary clinics that shared the source data for the study with VetCompass and was included in the analysis only to account for possible clustering within these veterinary groups.

One-year period prevalence values with 95% confidence intervals (CIs) described the probability of diagnosis at least once during 2019. CI estimates were derived from SEs based on approximation to the binomial distribution.^34^ The Mann–Whitney U-test was used to evaluate binary categorical variables for association with continuous variables.^34^ Demographic risk factor analysis used binary logistic regression modelling to evaluate univariable associations between risk factors (‘purebred’, ‘breed’, ‘adult body weight’, ‘age’, ‘sex–neuter’ and ‘veterinary group’) and periodontal disease in 2019. Risk factors with liberal associations in univariable modelling (*P* <0.2) were taken forward for multivariable evaluation. Model development used manual backwards stepwise elimination. Pair-wise interaction effects were evaluated for biological significance in the final model.^25^ Model fit was assessed using the area under the receiver operating characteristic (ROC) curve.^25^ Statistical significance was set at *P* <0.05.

Comorbid risk factor analysis individually evaluated each of the 40 most common other disorders within the sample cats against periodontal disease. Each comorbid disorder analysis used a separate multivariable binary logistic regression model that included the comorbid disorder of interest along with a fixed set of covariables included to account for confounding (‘purebred status’, ‘adult body weight’, ‘age’, ‘sex–neuter’ and ‘veterinary group’). Decision-making on which variables to include in these standard models used an ‘information theory’ approach to include a priori variables that the authors considered as potential confounders.^35^ Only the results for the comorbid factor of main interest are reported from each regression model. Statistical significance was set at *P* <0.05.

## Results

### Periodontal disease: frequency

From an available population of 1,255,130 cats under veterinary care during 2019, the study included a random sample of 18,249 (1.4%) cats. The sampled cats included 9141 (50.1%) females and 8944 (49.0%) males. The median age of the sampled cats was 5.67 years (interquartile range [IQR] 2.39–10.32, range 0.03–23.90). The median adult body weight of the sampled cats was 5.50 kg (IQR 3.99–7.40, range 1.50–15.00). Data completeness for the demographic variables in the sample was breed 98.7%, sex–neuter 99.1%, age 98.3% and adult body weight 61.5%.

From the sample of 18,249 cats, 3870 cats had recorded dental disease of any type across a range of diagnoses during 2019, giving an overall 1-year period prevalence of dental disease of 21.2% (95% CI 20.62–21.81). In total, 2780 cats met the case definition for periodontal disease during 2019, giving a 1-year period prevalence of 15.2% (95% CI 14.72–15.76). Periodontal disease was the most commonly recorded specific disorder in cats in 2019. Individual breeds with the highest 1-year period prevalence for periodontal disease included Siamese (18.7%, 95% CI 12.24–26.72) and Maine Coon (16.67%, 95% CI 11.37–23.18) (Table 1). The median age of cats with periodontal disease (9.47 years, IQR 5.96–12.97, range 0.04–23.38) was statistically higher than for cats without periodontal disease (4.94 years, IQR 1.95–9.51, range 0.03–23.90; *P* <0.001). The median body weight of cats with periodontal disease (5.67 kg, IQR 4.23–7.30, range 1.50–15.00) was statistically higher than for cats without periodontal disease (5.45 kg, IQR 3.92–7.43, range 1.50–15.00; *P* = 0.007).

**Table 1:** Prevalence of periodontal disease recorded in common cat breeds under primary care veterinary care at UK practices participating in the VetCompass programme from 1 January to 31 December 2019

Breed	Number	Periodontal disease	95% CI
Siamese	123	23 (18.7)	12.24–26.72
Maine Coon	168	28 (16.7)	11.37–23.18
British Shorthair	542	84 (15.5)	12.55–18.82
Crossbred	15,841	2442 (15.4)	14.86–15.99
Other	897	132 (14.7)	12.46–17.21
Persian	131	19 (14.5)	8.96–21.72
Bengal	219	21 (9.6)	6.03–14.28
Ragdoll	328	31 (9.4)	6.51–13.15
Total	18,249	2780 (15.2)	14.72–15.76

Data are presented as n (%) unless otherwise stated

CI = confidence interval

### Periodontal disease: risk factor analysis

All variables except purebred status were liberally associated with periodontal disease in univariable logistic regression modelling and were therefore evaluated using multivariable logistic regression modelling (Table 2). The final demographic multivariable model retained four risk factors: adult (body weight (kg); age (years), sex–neuter status; and veterinary group (Table 3). No biologically significant interactions were identified in the final model. The final model showed acceptable discrimination (area under the ROC curve 0.727). After accounting for the effects of the other variables evaluated, cats with adult body weights from 4.0 to <7.0 kg had higher odds of periodontal disease than cats weighing <3 kg. The odds of periodontal disease rose steeply with age before plateauing from 12 years of age onward. Neutered males had higher odds of periodontal disease than entire females. There was some evidence of differing odds of diagnosis with periodontal disease between the six veterinary groups included in the analysis.

**Table 2:** Descriptive and univariable logistic regression results for demographic risk factors for periodontal disease in cats (n = 18,249) under primary veterinary care in the VetCompass programme in the UK

Variable	Category	Case no. (%)	Non-case no. (%)	OR	95% CI	Category *P* value	Variable *P* value
Purebred	Crossbred	2442 (15.4)	13,399 (84.6)	Base			0.479
	Purebred	321 (14.8)	1843 (85.2)	0.96	0.84–1.08	0.481	
Breed	Crossbred	2442 (15.4)	13,399 (84.6)	Base			0.013
	Bengal	21 (9.6)	198 (90.4)	0.58	0.37–0.91	0.019	
	British Shorthair	84 (15.5)	458 (84.5)	1.01	0.79–1.27	0.958	
	Maine Coon	28 (16.7)	140 (83.3)	1.10	0.73–1.65	0.655	
	Persian	19 (14.5)	112 (85.5)	0.93	0.57–1.52	0.773	
	Ragdoll	31 (9.4)	297 (90.5)	0.57	0.39–0.83	0.003	
	Siamese	23 (18.7)	100 (81.3)	1.26	0.80–1.99	0.317	
	Other	132 (14.7)	765 (85.3)	0.95	0.78–1.14	0.572	
Adult (>9 months) body weight (kg)	<3.0	188 (15.7)	1009 (84.3)	Base			<0.001
	3.0 to <4.0 kg	280 (17.3)	1336 (82.7)	1.12	0.92–1.38	0.254	
	4.0 to <5.0	371 (19.9)	1497 (80.1)	1.33	1.10–1.61	0.004	
	5.0 to <6.0	405 (22.8)	1370 (77.2)	1.59	1.31–1.92	<0.001	
	6.0 to <7.0	343 (23.7)	1107 (76.3)	1.66	1.37–2.03	<0.001	
	7.0 to <8.0	219 (20.5)	851 (79.5)	1.38	1.11–1.71	0.003	
	8.0 to <9.0	142 (20.1)	563 (79.9)	1.35	1.06–1.72	0.014	
	9.0 to <11.0	162 (19.3)	678 (80.7)	1.28	1.02–1.62	0.035	
	⩾11.0	116 (16.2)	596 (83.7)	1.04	0.81–1.34	0.735	
	Unavailable	554 (7.9)	6,462 (92.1)	0.46	0.39–0.55	<0.001	
Age (years)	<3.0	202 (3.6)	5353 (96.4)	Base			<0.001
	3 to <6	494 (13.1)	3278 (86.9)	3.99	3.37–4.73	<0.001	
	6 to <9	605 (19.9)	2441 (80.1)	6.57	5.56–7.76	<0.001	
	9 to <12	601 (25.1)	1793 (74.9)	8.88	7.51–10.51	<0.001	
	12 to <15	451 (26.7)	1240 (73.3)	9.64	8.07–11.51	<0.001	
	⩾15	405 (27.2)	1084 (72.8)	9.90	8.26–11.87	<0.001	
	Unavailable	22 (7.3)	280 (92.7)	2.08	1.32–3.29	0.002	
Sex–neuter status	FE	336 (9.5)	3191 (90.5)	Base			<0.001
	FN	1012 (18.0)	4602 (82.0)	2.09	1.83–2.38	<0.001	
	ME	321 (9.8)	2937 (90.1)	1.04	0.88–1.22	0.650	
	MN	1094 (19.2)	4592 (80.8)	2.26	1.99–2.58	<0.001	
Veterinary group	1	615 (17.1)	2976 (82.9)	Base			<0.001
	2	7 (41.2)	10 (58.8)	3.39	1.28–8.93	0.014	
	3	797 (17.7)	3714 (82.3)	1.04	0.92–1.17	0.523	
	4	754 (12.4)	5308 (87.6)	0.69	0.61–0.77	<0.001	
	5	150 (24.0)	476 (76.0)	1.52	1.24–1.87	<0.001	
	6	457 (13.3)	2985 (86.7)	0.74	0.65–0.84	<0.001	

OR = odds ratio; CI = confidence interval; FE = female entire; FN = female neutered; ME = male entire; MN = male neutered

**Table 3:** Final multivariable logistic regression model for demographic risk factors associated with periodontal disease in cats (n = 18,249) under primary veterinary care in the VetCompass programme in the UK

Variable	Category	OR	95% CI*	*P* value
Adult (>9 months) body weight (kg)	<3.0	Base		
	3.0 to <4.0	1.08	0.88–1.33	0.453
	4.0 to <5.0	1.25	1.03–1.53	0.025
	5.0 to <6.0	1.37	1.12–1.67	0.002
	6.0 to <7.0	1.48	1.20–1.81	<0.001
	7.0 to <8.0	1.23	0.99–1.54	0.067
	8.0 to <9.0	1.27	0.99–1.63	0.059
	9.0 to <11.0	1.27	1.00–1.61	0.052
	⩾11.0	1.12	0.86–1.45	0.394
	Unavailable	0.6	0.50–0.72	<0.001
Age (years)	<3.0	Base		
	3 to <6	3.18	2.67–3.79	<0.001
	6 to <9	4.93	4.13–5.87	<0.001
	9 to <12	6.71	5.62–8.01	<0.001
	12 to <15	7.36	6.11–8.86	<0.001
	⩾15	7.89	6.54–9.52	<0.001
	Unavailable	2.92	1.83–4.64	<0.001
Sex–neuter status	FE	Base		
	FN	1.11	0.96–1.28	0.145
	ME	1.11	0.94–1.32	0.212
	MN	1.27	1.10–1.46	0.001
	Unavailable	0.99	0.58–1.70	0.980
Veterinary group	1	Base		
	2	3.61	1.27–10.24	0.016
	3	0.94	0.83–1.06	0.285
	4	0.99	0.88–1.12	0.898
	5	1.82	1.47–2.26	<0.001
	6	0.85	0.74–0.98	0.023

OR = odds ratio; CI = confidence interval; FE = female entire; FN = female neutered; ME = male entire; MN = male neutered

### Comorbid disorders risk factor analysis

Cats with periodontal disease had a higher median count of comorbid disorders per individual cat (3, IQR 2–4, range 1–14) than cats without periodontal disease (1, IQR 0–2, range 0–15; *P* <0.001 [note: this disorder count did not include periodontal disease itself]). After accounting for confounding by purebred status, adult body weight, age, sex–neuter status and veterinary group using multivariable analysis, cats with periodontal disease had 1.79 times the odds (95% CI 1.62–1.99, *P* <0.001) of diagnosis with at least one other disorder compared with cats without periodontal disease. Of the 40 other commonly diagnosed disorders in cats, 21 (52.5%) were positively associated with diagnosis of periodontal disease, four (10.0%) were negatively associated with diagnosis of periodontal disease and 15 (37.5%) showed no association with a diagnosis of periodontal disease (Table 4). The disorders with the highest associations with periodontal disease included cardiac dysrhythmia (odds ratio [OR] 2.32, 95% CI 1.70–3.18, *P* <0.001), aural discharge (OR 2.31, 95% CI 1.58–3.39, *P* <0.001) and hairball/furball (OR 2.31, 95% CI 1.57–3.41, *P* <0.001). After adjusting for confounding, the disorders with the lowest associations with periodontal disease included road traffic accident (OR 0.34, 95% CI 0.15–0.78, *P* = 0.011) and traumatic injury (OR 0.40, 95% CI 0.17–0.93, *P* = 0.034).

**Table 4:** Comorbid associations with periodontal disease for the 40 most common disorders recorded in cats (n = 18,249) under primary care veterinary care at UK practices participating in the VetCompass programme from 1 January to 31 December 2019

Comorbid disorder	OR	95% CI	*P* value
Cardiac dysrhythmia	2.32	1.70–3.18	<0.001
Aural discharge	2.31	1.58–3.39	<0.001
Hairball, furball	2.31	1.57–3.41	<0.001
Cat flu	2.18	1.42–3.35	<0.001
Haircoat disorder	2.06	1.68–2.53	<0.001
Thin	2.05	1.63–2.57	<0.001
Hyperthyroidism	2.02	1.61–2.54	<0.001
Heart murmur	2.01	1.71–2.36	<0.001
Chronic kidney failure	1.92	1.52–2.44	<0.001
Obesity/overweight	1.83	1.64–2.05	<0.001
Otitis externa	1.80	1.27–2.56	0.001
Conjunctivitis	1.69	1.25–2.29	0.001
Osteoarthritis	1.68	1.28–2.21	<0.001
Ocular discharge	1.66	1.05–2.64	0.031
Vomiting	1.65	1.34–1.99	<0.001
Dental disease (non-periodontal)	1.63	1.44–1.84	<0.001
Sneezing	1.55	0.99–2.43	0.055
Overgrown nail(s)	1.50	1.27–1.77	<0.001
Flea infestation	1.46	1.20–1.76	<0.001
Diarrhoea	1.45	1.14–1.86	0.003
Weight loss	1.42	1.19–1.70	<0.001
Anorexia	1.41	1.08–1.85	0.012
Inappropriate urination	1.38	0.84–2.25	0.206
Overgrooming	1.35	0.93–1.94	0.112
Urinary tract infection	1.17	0.73–1.86	0.511
Alopecia	1.14	0.67–1.93	0.624
Cat bite	1.12	0.78–1.62	0.526
Scab lesion(s)	1.12	0.68–1.83	0.665
Constipation	1.11	0.75–1.65	0.599
Flea bite hypersensitivity	1.07	0.77–1.47	0.695
Cystitis	1.06	0.74–1.52	0.743
Postoperative wound	1.04	0.62–1.73	0.879
Lameness	1.03	0.70–1.52	0.868
Poor quality of life	1.03	0.70–1.52	0.974
Allergic skin disorder	0.88	0.52–1.50	0.648
Abscess	0.78	0.62–1.00	0.048
Wound	0.74	0.54–1.02	0.065
Disorder undiagnosed	0.45	0.30–0.67	<0.001
Traumatic injury	0.40	0.17–0.93	0.034
Road traffic accident	0.34	0.15–0.78	0.011

Each association accounted for confounding effects from purebred status, adult body weight, age, sex–neuter and veterinary group

OR = odds ratio; CI = confidence interval

## Discussion

This study identified periodontal disease as the most common specific diagnosis in cats under primary veterinary care in the UK, with a 1-year period prevalence of 15.2%. This corresponds to a previously reported prevalence of 13.9% from a similar retrospective analysis of primary care clinical records,^1^ and largely confirms periodontal disease as a leading health issue in cats.^36^ Despite this, veterinary primary care levels of diagnosis are still much lower than some prospective studies that evaluated for periodontal disease during anaesthetised oral examinations and used radiography to report that up to 96% of cats show some form of dental disease.^6,7^ Consequently, despite the apparently high levels of periodontal disease diagnosis found in the current study, it is probable that a substantial proportion of cats with true periodontal disease still go undiagnosed or unrecorded in current primary veterinary care. Reasons for such underdiagnosis could include problematic patient temperament that can prohibit conscious clinical examination in some cats,^37–39^ clinical signs from oral disease rarely being specific to the oral cavity,^40^ inherent limitations to conscious oral examination in cats needed to identify periodontal disease,^4,41,42^ time constraints imposed during primary care consultations^43^ and even diminished veterinary motivation over time toward repeated formal diagnosis of recurring common disorders.^44,45^ It is also possible that there are wide differences in the clinical interest and confidence of individual veterinarians in dealing with dental cases in cats, with >50% of final-year veterinary students at UK universities reporting that they lack confidence in discussing orodental problems or performing a detailed examination of the oral cavity.^46^ Therefore, enhanced veterinary undergraduate and postgraduate education on dental care has the potential to substantially improve the detection rates of periodontal disease in cats.

In the current study, no significant association was seen between purebred/crossbred status and diagnosis with periodontal disease. This is in contrast to some earlier studies that reported that purebred cats had significantly worse gingivitis scores^7^ and increased severity of periodontitis^6^ compared with crossbred cats. Unfortunately, formalised measures of the severity of periodontal disease were not extracted during the current VetCompass study and therefore it was not possible to explore differential severity risk between purebred and crossbred cats. However, the current study did identify some individual breeds at differential disease risk. Breeds with the highest 1-year period prevalence for periodontal disease in the current study included Siamese (18.7%) and Maine Coon (16.7%) cats. A study of colony cats sharing the same environment and fed the same diet reported a higher risk of periodontal disease for Burmese, Bengal, Somali, Maine Coon, Bobtail and Abyssinian cats, although that study was limited by the small sample size for each breed and high familial relatedness of some individuals.^7^ Nonetheless, it appears that breed and therefore genetics play some role in periodontal disease development in cats. Such a genetic influence is supported by parallel breed-related differential risks reported for periodontal disease in dogs.^33,44,47–50^ Racial and ethnic differences in periodontal disease pro-clivity in humans further support potential general genetic influences on periodontal disease across species.^51,52^ Such genetic effects may be mediated by differing cytokine activity,^53^ but the multifactorial aetiology of periodontal disease with interactions between manifold genetic and environmental factors is likely to make full elucidation of causal pathways challenging to achieve. It is possible that companion animals could offer a useful model for human translational research on periodontal disease.^54^ Increasing awareness of differential breed-related risks for periodontal disease in cats offers more immediate benefits of potentially targeted dental care for high-risk breeds and demographics within these breeds to reduce periodontal disease development and severity.

The median age of cats with periodontal disease in the current study was substantially older than for cats not diagnosed with periodontal disease (9.47 vs 4.94 years). After accounting for other confounding variables, age was the strongest predictor of periodontal disease, with the final multivariable model showing a progressive increase in risk with ageing; cats aged >9 years had more than six times the odds of periodontal disease compared with cats aged <3 years. An important role of ageing on periodontal disease development in cats is supported by similar results from a radiographic study of colony cats^7^ but is contrary to another radiographic study of cases referred to a US tertiary care centre that identified young cats with similarly high prevalence values for some forms of periodontitis to the general population on thorough dental examination, although severity varied with age.^6^ Increasing age is generally accepted as a major risk factor for periodontal disease in humans.^55^ Overall, ageing does appear to be an important risk factor for the increasing presence and severity of periodontal disease in cats. This awareness should encourage heightened vigilance in primary care veterinarians for periodontal disease in older cats and perhaps to consider greater promotion of routine screening or wellness programmes with a focus on dental health.^56^ The interesting finding in the current study that periodontal disease risk plateaus once cats reach 12 years of age could perhaps reflect a negative effect that moderate or severe periodontitis may have on the life expectancy of these cats and might lend further support to the clinical value of improved detection and earlier intervention when treating periodontal disease in older cats.

The median body weight of cats with periodontal disease (5.67 kg) was statistically higher than for cats without periodontal disease (5.45 kg); however, biologically, this equated to less than a quarter of a kilogram difference in the median values. In the multivariable analysis, body weight categories of cats weighing >4 kg tended to show 1.25 times or greater odds of periodontal disease than cats weighing <4 kg. In the current study, body weight was calculated as the average (mean) of all recorded adult body weights of each cat over its available lifetime clinical records and therefore should be interpreted as reflecting overall lifetime adult body weight rather than body weight at the point of diagnosis. This finding of increased risk of periodontal disease in heavier cats is supported by previous research that reported larger cats being at higher risk than smaller cats.^57^ In contrast, an association in the opposite direction between periodontal disease and body weight has been reported in several studies in dogs. Using a data source and study design similar to the current study, dogs weighing <10 kg had over three times the odds of periodontal disease than dogs weighing 30–40 kg,^33^ with similar findings reported by others.^14,58–60^ The broad phenotypic diversity that humans have selected across the spectrum of current dog breeds, with breed-typical body weights ranging almost 100-fold, suggest that a periodontal disease predisposition truly does exist for smaller types of dogs.^61,62^ However, the more limited phenotypic variation in domestic cats makes it less clear whether the current results indicate true predisposition in larger types of cats, given that these results could also just reflect effects from varying levels of adiposity in cats.^14,58–60^ This latter explanation is supported by findings that many cats with early periodontal disease maintain a normal food intake despite some oral discomfort,^57^ although cats with severe periodontitis are shown to have increased pain scores and decreased food intake vs cats with minimal periodontal disease.^10,63^

Following multivariable analysis, cats with periodontal disease had 1.79 times the odds of having at least one other disorder recorded compared with cats without a periodontal disease diagnosis. It is possible that some, or even all, of the health differences detected in the current study are explainable by diagnostic access bias. Diagnostic access bias describes a systematic differential probability of diagnosis of further disorders in individuals that already have access to disorder testing and detection for an initial disorder.^64^ Therefore, higher odds of having at least one other disorder recorded in cats with periodontal disease could simply reflect enhanced levels of diagnostic care being given to cats with an existing periodontal disease diagnosis vs other cats.^65^ However, the case for true links between periodontal disease and reduced overall health in cats is strengthened by the substantial body of evidence that supports poorer health in dogs with periodontal disease.^19–22,66–71^ In addition, markers of systemic inflammation are reported to increase in cats with periodontal disease and then to decrease once periodontal disease is successfully treated, suggesting that a chronic inflammatory response exists in cats to periodontal disease that could have a generally negative impact on feline health.^57^ Evidence is now starting to emerge in the human literature that also demonstrates a direct correlation between systemic disease and periodontitis.^72^ Delving more deeply into associations between periodontal disease and wider health, an issue that remains speculative in cats is the direction of any causality of health effects from periodontal disease. The cross-sectional design of the current study precludes inference on the temporality of the development of periodontal disease compared with these other comorbid disorders so the current study leaves it equally open that periodontal disease could promote poorer general health, or that poorer general health could promote periodontal disease, or both. Either way, the current results do suggest the value of greater clinical vigilance for comorbidity in cats diagnosed with periodontal disease.

To give a clearer picture on which comorbidities were specifically linked with periodontal disease in cats, the current study explored comorbid associations between periodontal disease and the 40 other most common disorders recorded in the cats. Of these 40 common disorders, 21 (52.5%) were more likely to be comorbidly diagnosed in cats with periodontal disease. Individual disorders with the highest comorbid associations with periodontal disease included cardiac dysrhythmia (OR 2.32), aural discharge (OR 2.31) and hairball/furball (OR 2.31). In humans, multiple comorbidities are commonly reported between chronic inflammatory diseases such as periodontal disease and other disorders.^73,74^ From an aetiopathogenetic perspective, this is not unexpected as many inflammatory diseases share common causal factors, including immune pathways, genetic and epigenetic risk factors.^74,75^ So, it is unsurprising that similar results were found in the current paper where cats with periodontal disease had an average of three other comorbid disorders.

Based on studies in dogs,^19,20,22^ the current study hypothesised that cats with recorded periodontal disease have a higher probability of renal and cardiac conditions as comorbid diagnoses. The results support both hypotheses, with cats with periodontal disease showing 1.92 times the odds of chronic kidney failure, as well as 2.32 times the odds of cardiac dysrhythmia and 2.01 times the odds of heart murmur. In dogs, renal diseases, such as pyelonephritis and glomerulonephritis, can follow of a low-grade bacteraemia and toxaemia that has been associated with periodontal disease.^19,21^ Periodontopathogenic bacteria appear to have an affinity for renal endothelium, leading to immune complex-mediated damage.^19,76^ CKD can result from such damage, with many studies suggesting that lesions tend to develop from chronic or persistent insult to the kidneys.^19,21^ Cardiac conditions, such as endocarditis, can also be attributed by the low-grade bacteraemia.^21^ However, in humans, a pleiotropy phenomenon has been proposed between periodontal disease and cardiovascular diseases, suggesting that periodontal disease is not causally related to cardiovascular disease, but rather that both are a result of a common inflammatory pathway.^74,77^

Comorbid associations uncovered by large-scale epidemiologic analyses such as the current study are recognised as useful guides for population-based clinical health and therapeutic efforts, as well as for positing new aetiopathogenic pathways and pathophysiological mechanisms that link these disorders.^78^ Studies reporting associations between periodontal and specific systemic diseases in dogs have supported several new discoveries on disease causation.^19–22,66–71^ The transient bacteraemia induced by mastication was once thought to contribute to distant effects seen in other organs, although recent human studies have shown no significant differences in bacterial community profiles in blood from participants with and without periodontal disease, and only marginal increases in bacterial levels.^79^ Consequently, it is likely that mechanisms other than direct bacteraemia play the dominant roles in increased systemic disease in periodontal disease patients. More recent hypotheses explain that systemic spread of bacterial endotoxins, as well as inflammatory mediators and cytokines, from acute-phase inflammatory responses to periodontal pathogens can have serious negative impacts on the vascular system throughout the body.^19,69,77,80^ Stronger evidence for the role of periodontal disease as a causal agent for reduced overall health could come from studies that show a reversal of health issues following successful management of periodontal disease. While some intervention studies in dogs have attempted to show that the treatment of periodontitis leads to an improvement in the health of other organ disease,^81,82^ there are no similar intervention or association studies testing or demonstrating such effects for felines. Such studies in cats would be useful to evaluate whether the early management of periodontal disease or preventative care/wellness programmes/senior cat clinics are associated with a decreased incidence of comorbidities or disease progression.

This study had some limitations in addition to those that have been previously reported in the application of primary care veterinary clinical records for research.^32,83^ As mentioned above, the current study did not extract information on the severity of periodontal disease, thereby precluding analysis of risk factors for varying levels of severity. The statistical power to detect and report associations varied across the comorbid disorders in line with the varying prevalence of these comorbid disorders.^84^ The current study explored multiple analyses and so there is a risk of false positive findings (type I error) being reported.^85^ Consequently, individual results reported here should be considered as exploratory ‘hypothesis-generating’ rather than ‘hypothesis-confirmatory’. The statistical findings of the current study should be incorporated with the results of other studies and combined with our own individual understanding of periodontal disease, to optimise our unique interpretations. In primary care practice, periodontal disease is predominantly diagnosed based on a visual oral assessment of the conscious animal and therefore the true prevalence of periodontal disease in cats is likely to have been heavily underestimated in the current study.^15^ The clinical distinction between the presence of calculus/tartar and periodontal disease may be blurred in the primary care setting, and therefore it is possible that some misclassification occurred at this level of diagnostic precision.

## Conclusions

This study has identified periodontal disease as the most commonly diagnosed specific disorder in cats under primary veterinary care in the UK and largely confirms periodontal disease as a leading health issue in cats. Ageing is identified as the strongest predictor of periodontal disease risk in cats, suggesting the potential for important health gains from greater emphasis on dental care and health in cats as they age. The study offers evidence on a wide range of comorbidities with periodontal disease in cats that suggests a close link between periodontal disease and reduced overall health in cats. These results also flag the growing value of big data resources such as VetCompass to increase our overall understanding of feline health at a population level.
